# Arterial Hypertension Aggravates Innate Immune Responses after Experimental Stroke

**DOI:** 10.3389/fncel.2015.00461

**Published:** 2015-11-27

**Authors:** Karoline Möller, Claudia Pösel, Alexander Kranz, Isabell Schulz, Johanna Scheibe, Nadine Didwischus, Johannes Boltze, Gesa Weise, Daniel-Christoph Wagner

**Affiliations:** ^1^Fraunhofer Institute for Cell Therapy and ImmunologyLeipzig, Germany; ^2^Institute of Anatomy, Histology and Embryology, University of LeipzigLeipzig, Germany; ^3^Research Group Human Biology, Institute of Biology, University of LeipzigLeipzig, Germany; ^4^Fraunhofer Research Institution for Marine Biotechnology and Institute for Medical and Marine Biotechnology, University of LübeckLübeck, Germany; ^5^Department of Neurology, University of LeipzigLeipzig, Germany

**Keywords:** stroke, hypertension, animal model, inflammation, neutrophils, macrophages, adhesion molecules, chemokines

## Abstract

Arterial hypertension is not only the leading risk factor for stroke, but also attributes to impaired recovery and poor outcome. The latter could be explained by hypertensive vascular remodeling that aggravates perfusion deficits and blood–brain barrier disruption. However, besides vascular changes, one could hypothesize that activation of the immune system due to pre-existing hypertension may negatively influence post-stroke inflammation and thus stroke outcome. To test this hypothesis, male adult spontaneously hypertensive rats (SHRs) and normotensive Wistar Kyoto rats (WKYs) were subjected to photothrombotic stroke. One and 3 days after stroke, infarct volume and functional deficits were evaluated by magnetic resonance imaging and behavioral tests. Expression levels of adhesion molecules and chemokines along with the post-stroke inflammatory response were analyzed by flow cytometry, quantitative real-time PCR and immunohistochemistry in rat brains 4 days after stroke. Although comparable at day 1, lesion volumes were significantly larger in SHR at day 3. The infarct volume showed a strong correlation with the amount of CD45 highly positive leukocytes present in the ischemic hemispheres. Functional deficits were comparable between SHR and WKY. Brain endothelial expression of intercellular adhesion molecule 1 (ICAM-1), vascular cell adhesion molecule 1 (VCAM-1), and P-selectin (CD62P) was neither increased by hypertension nor by stroke. However, in SHR, brain infiltrating myeloid leukocytes showed significantly higher surface expression of ICAM-1 which may augment leukocyte transmigration by leukocyte–leukocyte interactions. The expression of chemokines that primarily attract monocytes and granulocytes was significantly increased by stroke and, furthermore, by hypertension. Accordingly, ischemic hemispheres of SHR contain considerably higher numbers of monocytes, macrophages and granulocytes. Exacerbated brain inflammation in SHR may finally be responsible for larger infarct volumes. These findings provide an immunological explanation for the epidemiological observation that existing hypertension negatively affects stroke outcome and mortality.

## Introduction

About one-third of the world population suffers from arterial hypertension, defined by blood pressure values higher than 140/90 mmHg (James et al., 2014). Among all organs, the brain is most susceptible to increased blood pressure. Without perceptible symptoms, hypertension successively remodels the brain microvasculature, ultimately causing parenchymal damage, cognitive impairment and dementia (Pantoni, 2010). Moreover, hypertension-related sclerosis of extra- and intracerebral arteries is a leading cause for stroke, whereas existing hypertension correlates with worse outcome and increased mortality after stroke (Willmot et al., 2004; O’Donnell et al., 2010). A disturbed autoregulatory capacity of brain vessels, endothelial oxidative stress, and focal hypertensive blood–brain barrier damage may contribute to increased brain injury after stroke (Faraco and Iadecola, 2013). However, the role and impact of the immune system in this pathophysiological cascade is poorly understood.

It is well-recognized though that hypertension and hypertensive brain damage are determined and maintained by a plethora of inflammatory processes. Increased blood pressure causes an activation of brain-resident astrocytes and microglia and an upregulation of adhesion molecule (AM) expression on brain endothelial cells (Gouw et al., 2011; Wardlaw et al., 2013; Kaiser et al., 2014). In the periphery, hypertension correlates with monocyte activation (Dorffel et al., 1999; Ishibashi et al., 2004) and increased plasma levels of pro-inflammatory cytokines (Kim et al., 2008). Autonomic dysfunction during hypertension (Mancia and Grassi, 2014) promotes myelopoiesis and leukocyte activation in secondary lymphatic organs (Ganta et al., 2005; Harwani et al., 2012; Zubcevic et al., 2014). Chronic hypertension thus perpetuates a state of inflammation and immune activation that may affect severity and progression of acute stroke.

In fact, stroke itself is characterized by a sterile tissue inflammation initiated by damage-associated molecular patterns and cytokine release (Shichita et al., 2012b). In the acute phase, post-stroke inflammation is dominated by innate immune cells, namely polymorphonuclear neutrophils (PMNs), monocytes, macrophages and microglia (Gelderblom et al., 2009; Benakis et al., 2014; Moller et al., 2014). Importantly, it has been shown that less PMN infiltration and the correct as well as timely resolution of inflammation by macrophages are important indicators for better stroke outcome (Gliem et al., 2012; Neumann et al., 2015).

We hypothesized that the aforementioned immunological sequelae of existing hypertension could dysregulate and exacerbate the immune response to stroke and thus affect the overall outcome. To investigate this hypothesis, we compared stroke development, cerebral AM expression and neuroinflammation in spontaneously hypertensive rats (SHRs) and normotensive Wistar Kyoto rats (WKYs). Due to cerebral microangiopathy and impaired cerebral blood flow regulation, traditional stroke models such as transient or permanent middle cerebral artery occlusion (MCAO) generally cause larger infarcts in hypertensive rats (Hom et al., 2007; De Geyter et al., 2012; Kang et al., 2014). This would, however, confound the investigation of secondary immune-related changes. To dissect the effect of hypertension on post-stroke inflammation we hence decided to use the photothrombotic stroke (PT) model (Watson et al., 1985), that allows generating an ischemic lesion independent of the status of the cerebrovascular system. In fact, we found that SHR and WKY exhibit comparable lesion volumes at 24 h after stroke. However, 2 days later, infarct volumes were significantly larger in SHR, possibly as consequence of increased amounts of myeloid innate immune cells that infiltrated into the ischemic brain. Our findings therefore offer an immunological explanation for the epidemiologically established fact that arterial hypertension negatively affects stroke outcome and mortality.

## Materials and Methods

### Animals and Group Allocation

All animal experiments were carried out according to the Guide for the Care and Use of Laboratory Animals published by the US National Institutes of Health (NIH Publication No. 85-23, revised 1996) and approved by the appropriate federal authority. SHR and WKY (Charles River, Sulzfeld, Germany) at the age of 12–14 weeks were randomly assigned to the following experimental groups: (1) naive WKY (*n* = 10); (2) naive SHR (*n* = 10); (3) WKY with stroke (*n* = 18); (4) SHR with stroke (*n* = 22). Group 4 was planned with additional four animals to compensate for a potentially increased stroke-related mortality in hypertensives. Animals were randomly assigned to subgroup analyses as specified in the according methods sections. Health status and body weight was monitored twice a day during the experiment. Exclusion criteria were: (i) weight loss of more than 20%; deterioration of the health status that requires supportive action; (ii) absence of a cortical lesion typical for the stroke model used; (iii) massive brain hemorrhage.

### Experimental Stroke Model

Photothrombotic stroke was induced in groups 3 and 4 as described previously (Diederich et al., 2014). Briefly, rats were anesthetized with 2% isoflurane in 100% oxygen. Body temperature was maintained at 36.5 ± 0.5°C by a feedback-controlled heating device. PT was induced in the right frontal cortex. For illumination, a fiber-optic bundle of a cold light source (4.5 mm diameter) was centered stereotactically 1 mm posterior and 2.5 mm lateral from Bregma on the intact skull. Afterward, 0.3 mL of a sterile-filtered Rose Bengal solution (Sigma, Germany) was injected into the tail vein and the brain was illuminated for 20 min. After surgery, the skin was sutured and animals were placed in heated cages for recovery.

### Magnetic Resonance Imaging

Magnetic resonance imaging (MRI) investigations were performed at a 7 Tesla (T) PharmaScan 70/16 (Bruker Biospin, Germany). The system with a gradient strength of 375 mT/m was equipped with a transmit-only volume coil for excitation and a receive-only rat brain array coil for detection (Bruker Biospin, Ettlingen, Germany). All data were acquired with Topspin 2.0 and Paravision 5.1 software (Bruker BioSpin). Animals were anesthetized with 2% isoflurane in 100% oxygen. Body temperature was monitored via a rectal probe and maintained at 37.0 ± 0.5°C. The region of interest was localized using a standard gradient echo FLASH survey scan (Tripilot, Bruker). For acquisition of 22 axial slices, a spin echo multislice rapid acquisition and relaxation enhancement (RARE) technique was applied with the following parameters: repetition time (TR) = 3440 ms, echo time (TE) = 48.8 ms, matrix size (512 × 512), field of view = (27.5 × 27.5) mm^2^, slice thickness = 0.8 mm, slice gap = 0.2 mm. The corresponding voxel size was 0.05 mm × 0.05 mm × 0.8 mm. The total acquisition time was 18 min. Two measurements (day 1, *n* = 4/5; day 3, *n* = 8/9) were acquired, exported as DICOM files and processed with ImageJ (NIH, Bethesda, MD, USA). The area of the ischemic lesion (LV), the ipsilateral (SVi) and contralateral (SVc) side ventricles and both hemispheres (HVi and HVc) were manually segmented using anatomical landmarks and T2 hyperintense structures by an investigator blinded for the group allocation. These parameters were used to calculate the lesion volume and the space occupying effect due to brain edema (%HSE) as described previously (Gerriets et al., 2004).

### Behavioral Tests

Behavioral tests were carried out 1 and 3 days after stroke onset at the beginning of the dark phase (6:00 pm) by an investigator blinded to the group allocation (*n* = 7/10). The assessment of the modified neurological severity (mNS) score was performed as described previously (Chen et al., 2001). For the adhesive removal test (ART), adhesive labels of 64 mm^2^ were stuck on the ipsi- and contralateral palm of the forepaw. The time needed to remove the labels was assessed in technical duplicates and converted into the asymmetry score using the following formula: (removal time ipsilateral – removal time contralateral)/(removal time ipsilateral + removal time contralateral; Minnerup et al., 2011). Animals were adapted to the ART setting for 3 days prior to the start of the experiment.

### Sampling

At day 4 after stroke, rats were sacrificed by CO_2_ exposition subsequent to deep inhalation anesthesia (5% isoflurane in 100% oxygen). After opening of the thoracic cavity, 1 mL of anticoagulated blood was withdrawn from the left ventricle and collected in 2 mM EDTA. For flow cytometry or quantitative real-time PCR, rats were transcardially perfused with 200 mL of ice-cold PBS. Rats that were allocated to histological analyses were additionally perfused with 200 mL of ice-cold 4% formalin solution.

### Analysis of Peripheral Blood Leukocytes

Absolute counts of PMNs and monocytes were determined by an animal blood counter (scil Vet abc, SCIL animal care company GmbH, Viernheim, Germany; *n* = 4/4/7/10). To determine CD43 expression on monocyte subsets, 50 μl EDTA-blood was diluted in 50 μl phosphate buffered saline (PBS) and incubated with a mixture of the following monoclonal antibodies: CD43-Alexa Fluor 647 (clone W3/13, Biolegend, San Diego, CA, USA), CD45-APC-Cy7 (OX1, BD Biosciences, Heidelberg, Germany), CD11b-Pacific Blue (MRC-OX42, Abd Serotec, Oxford, UK) for 20 min at 4°C. Erythroid cells were lysed by short-term incubation with distilled water followed by repeated washing with FC buffer (PBS containing 3% fetal calf serum). Remaining leukocytes were resuspended in 300 μL FC buffer. Flow cytometric acquisition and analysis were performed using a 3-laser FACSCanto II equipped with the FACSDiva software (BD Biosciences) by an investigator blinded to the group allocation.

### Quantitative Real-time PCR

Brains hemispheres were removed (*n* = 3/3/3/3), separated and manually dissociated using razor blades. Total RNA of 100 mg tissue was extracted by homogenization in 1 mL Trizol using a ULTRA-TURRAX^®^ (Ika, Staufen, Germany) and further purified by RNeasy Mini Kit (Qiagen, Hilden, Germany). Single-strand cDNA copies were generated from 5 μg of purified total RNA by using Oligo(dT) 15 primers (Promega, Mannheim, Germany) and Superscript III reverse transcriptase (Invitrogen/Life Technologies, Darmstadt, Germany) according to manufacturer’s instructions. Quantification of mRNA expression was performed and monitored using an ABI 7900 real-time PCR system (Applied Biosystems, Darmstadt, Germany) applying the following conditions: initial denaturation at 95°C for 10 min, followed by 50 cycles at 95°C for 15 s and 55°C for 1 min. All qRT-PCR reactions were conducted in a total volume of 10 μL with addition of QuantiTect SYBR Green (Qiagen) and gene specific QuantiTect primers (Qiagen): VCAM-1 (QT00178500), ICAM-1 (QT00174447), CD62P (QT00180418), CCL2 (QT00183253), CCL3 (QT00378350), CCL4 (QT00187075), CCL7 (QT01593767), CXCL2 (QT00184891), CXCL5 (QT00392777), IL1-β (QT00181657), IL-6 (QT00182896), TNF-α (QT00178717), IL-10 (QT00177618) MMP9 (QT00178290) TGF-β (QT00187320). Data were analyzed using the relative standard curve method and normalized to the average cycle threshold of the housekeeping genes B2M (QT00176295), RPL13a (QT00425873), rpl22 (QT00385119), Yhwaz (QT02382184).

### Histology

Perfusion-fixed brains were removed, kept in 4% formalin solution for 24 h and treated with 30% sucrose solution for 3 days (*n* = 3/3/3/3). Subsequently, brains were shock-frozen and stored at -80°C until further use. Frozen brains were cut into 20 μm coronal slices and mounted on Superfrost Plus slides (Carl Roth, Karlsruhe, Germany). Next, brain slices were incubated in blocking buffer (5% goat serum, 0.3% Triton X-100 and PBS) for 2 h, followed by incubation with combinations of rabbit anti-glial fibrillary acidic protein (GFAP, 1:500, DAKO, Hamburg, Germany), rabbit anti-ionized calcium binding adaptor molecule 1 (Iba1, 1:200, Wako, Neuss, Germany), mouse anti-intercellular adhesion molecule 1 (ICAM-1/CD54, 1:200, Hölzel Diagnostika, Cologne, Germany), mouse anti- P-selectin (CD62P, 1:200, Novus Biologicals, Cambridge, UK) and biotinylated solanum tuberosum lectin (STL, 1:300, Linaris, Dossenheim, Germany) overnight at 4°C. Sections were then incubated with the according combinations of goat anti-mouse Alexa Fluor 488, goat anti-mouse Alexa Fluor 546, goat anti-rabbit Alexa Fluor 488, goat anti-rabbit Alexa Fluor 546 (all 1:400, Invitrogen, Darmstadt, Germany) and streptavidin (1:1000, Dianova, Hamburg, Germany) conjugated with Cy5 for 2 h at room temperature. All sections were counter-stained with 4′, 6-diamidin-2-phenylindol (DAPI; Sigma) using a concentration of 1 μg/mL for 10 min. As negative control, according brain slices were processed analogously except the use of the primary antibody. Fluorescence image stacks were acquired with a Zeiss (Göttingen, Germany) LSM710 confocal laser scanning microscope (Laser: Diode 405, Argon 488, Helium-Neon 543; Objective: Plan- Apochromat 63x/1.40 oil) and saved using a compression free image format. Image stacks were presented as maximum intensity projection or as orthogonal slice view.

### Flow Cytometric Analysis of Brain Endothelial Cells and Leukocytes

After transcardial perfusion with 200 mL of ice-cold PBS, brains were removed and separated from the cerebellum and olfactory bulb (*n* = 3/3/7/10). Brain hemispheres were hemisected and mechanically dissected using razor blades. Single cell suspensions of the ischemic and contralateral brain hemispheres were obtained by intermittent enzymatic digestion with collagenase I (Sigma, Munich, Germany) and DNAse I (Roche, Mannheim, Germany) in Hanks balanced salt solution (HBSS) for 45 min at 37°C, including two trituration steps using a gentleMACS Dissociator (Miltenyi Biotec., Bergisch Gladbach, Germany). Immune cells were then separated by density gradient centrifugation on discontinuous Percoll (GE Healthcare, München, Germany) gradients composed of four sequent layers (80%/38%/21% Percoll covered with cell culture medium). Cells accumulating in between 80%/38% Percoll were harvested and washed. Finally, cells were resuspended in 100 μL FC buffer and cell counts and viability were determined by trypan blue exclusion in a hemocytometer. Total cell suspensions were subsequently incubated with a specific FC-blocking reagent (purified anti-rat CD32; BD Bioscience) for 10 min at 4°C and labeled with the following anti-rat antibodies for 20 min at 4°C: CD163-FITC (clone ED2, Abd Serotec), CD62P-Alexa Fluor 488 (Psel.KO.2.12, Abd Serotec), Granulocytes-PE (RP1, BD Biosciences), CD3-PE (1F4, Biolegend), RT1B-PerCP (OX6, BD Biosciences), VCAM-1-PE (MR106, Abd Serotec), CD80-Biotin (3H5, Ebioscience, San Diego, CA, USA), ICAM-1 PerCP-eFluor 710 (1A29, Ebioscience), CD31-eFluor 660 (TLD-3A12, Ebioscience), CD45-APC-Cy7 (OX1, BD Biosciences), CD11b-Pacific Blue (MRC-OX42, Abd Serotec). Biotinylated CD80 was secondly labeled with streptavidin-PE-Cy7 (Ebioscience) for additional 15 min at 4°C in the dark. After final washing, cell suspensions were resuspended in 200 μL FC buffer and stored at 4°C until further use. Flow cytometric acquisition was performed as described above by an investigator blinded to the group allocation. Absolute numbers of brain immune cells were determined by additional Trucount Tube measurements (BD Biosciences). The median fluorescence intensity (MFI) of the AMs ICAM-1, VCAM-1, or CD62P was corrected by subtraction of the population specific median autofluorescence intensity.

### Statistics

Data were presented as mean (M) ± standard deviation (SD). Two experimental groups were compared by unpaired *t*-tests. Data from more than two groups were analyzed by one-way ANOVA followed by Newman–Keuls multiple comparison test. Functional data were analyzed by two-way repeated measures ANOVA followed by Bonferroni’s *post hoc* test. Correlation was determined by Pearson’s correlation coefficient analysis. P values of <0.05 were considered statistically significant. Statistical analyses were performed using GraphPad Prism 5.03.

## Results

### Exclusions

Two SHR and one WKY were excluded from the study due to the absence of an ischemic lesion. Two WKY and one SHR died during the MR measurement. Another SHR was excluded solely from the behavioral tests due to a lack of compliance. Owing to technical problems the brain of one SHR could not be processed for flow cytometry, and the brains of two SHR and one WKY could not be analyzed for the expression of AMs.

### Impact of Hypertension on Infarct Volume and Functional Deficits

**FIGURE 1:**
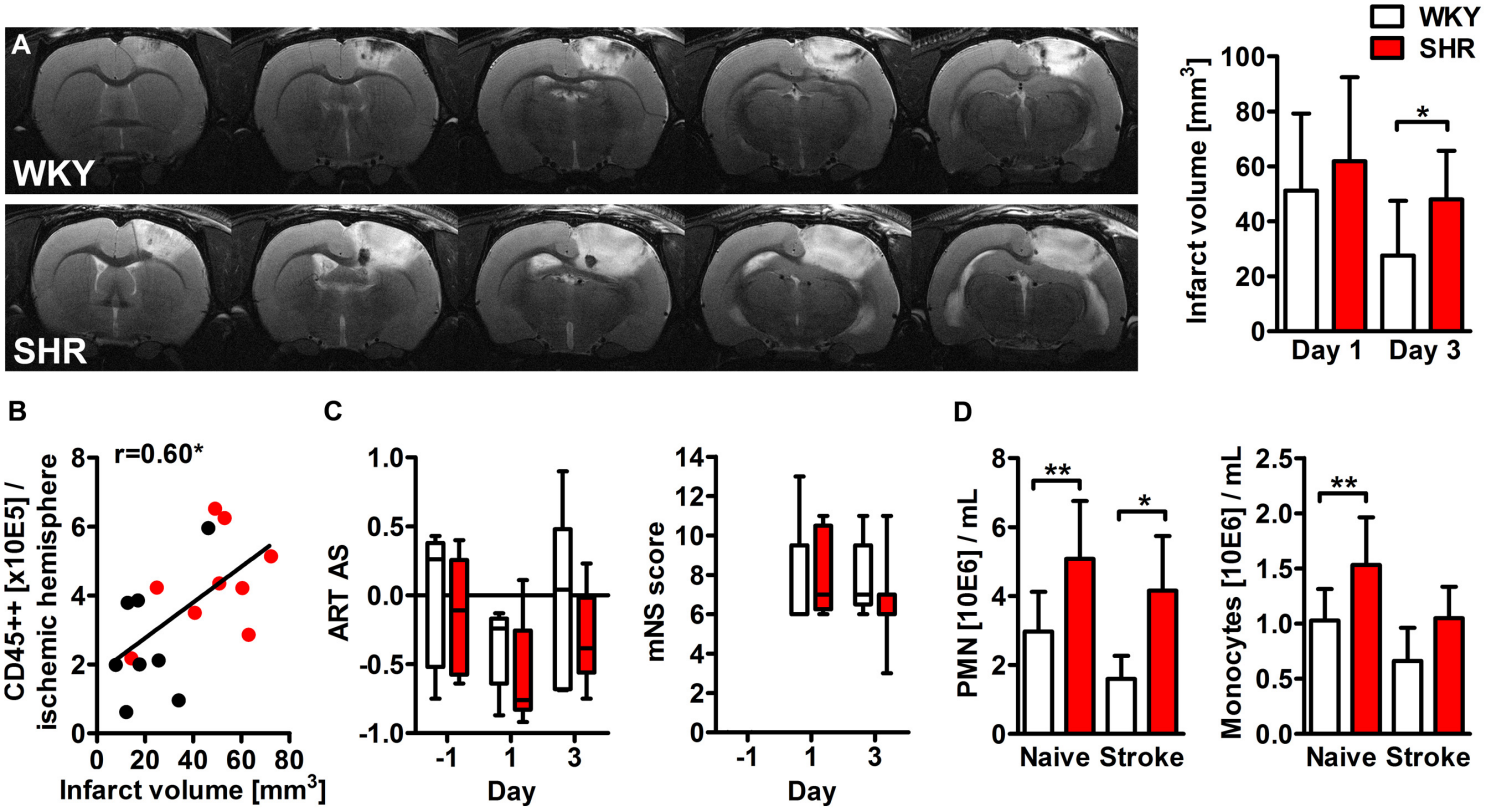
**Infarct volume, neurological deficits and myeloid blood cell counts after photothrombotic stroke (PT) in spontaneously hypertensive rats (SHRs) and Wistar Kyoto rats (WKYs). (A)** The infarct volume was determined by magnetic resonance (MR) imaging at days 1 and 3 after PT (*n* = 4/5/8/9). Images show representative series of MR images 3 days after PT in WKY and SHR. **(B)** Pearson’s correlation of the infarct volume and the amount of CD45 highly positive cells within the ischemic hemisphere (*n* = 8/9). **(C)** Neurological deficits were assessed 1 day before, and at days 1 and 3 after PT using the adhesive removal test (ART) asymmetry score (AS) and the modified neurological severity (mNS) score (*n* = 5/8). **(D)** Quantification of circulating polymorphonuclear granulocytes (PMN) and monocytes in peripheral blood samples (*n* = 4/4/5/9). Data are mean values ± standard deviation. ^∗^*p* < 0.05, ^∗∗^*p* < 0.01.

We first determined the impact of pre-existing hypertension on photothrombotic lesion development by MR imaging. All animals that met the inclusion criteria exhibited a hyperintense lesion within the right cortex. The calculation of the space-occupying effect revealed no differences between SHR and WKY at days 1 (6.8 ± 5.4 %HSE versus 5.9 ± 3.7 %HSE, *p* = 0.78) and 3 (5.4 ± 3.0 versus 4.9 ± 3.4, *p* = 0.75) after PT. The infarct volume was also comparable at day 1, but significantly less decreased in SHR 3 days after PT (**Figure 1A**). Pearson’s correlation test showed a strong relation between the infarct volume at day 3 and the amount of CD45 highly positive leukocytes within the ischemic hemisphere at day 4 (**Figure 1B**). Next, we investigated whether hypertension also affects functional outcome in the acute phase after stroke by means of the ART and the mNS scoring system. Both tests revealed a neurological deficit after stroke, but no significant differences between SHR and WKY (**Figure 1C**).

### Impact of Hypertension and Stroke on Circulating Immune Cells

Hematological analyses showed increased levels of circulating polymorphonuclear granulocytes (PMN) and monocytes already in naive SHR. PT had no statistically significant impact on circulating PMN or monocyte counts (**Figure 1D**). We next determined the impact of hypertension and stroke on the percentages of classical and non-classical monocytes by flow cytometric analysis of CD43 expression. CD43+ non-classical monocytes in rats correspond to Ly6C low/CX3CR1 high monocytes in mice whereas CD43- classical monocytes resemble Ly6C high/CCR2 high monocytes (Strauss-Ayali et al., 2007). Naive SHR showed a significantly higher proportion of CD43+ non-classical monocytes compared to WKY (M ± SD: 88.8 ± 7.8% versus 76.8 ± 3.1%, *p* < 0.05, *n* = 4/4). This relation was similar after PT (M ± SD: 85.8 ± 3.9% versus 77.9 ± 3.8%, *p* < 0.01, *n* = 5/9), indicating that circulating monocyte subsets were altered by arterial hypertension, but not by stroke.

### Cerebral AM Expression is Altered by Hypertension and Stroke

**FIGURE 2:**
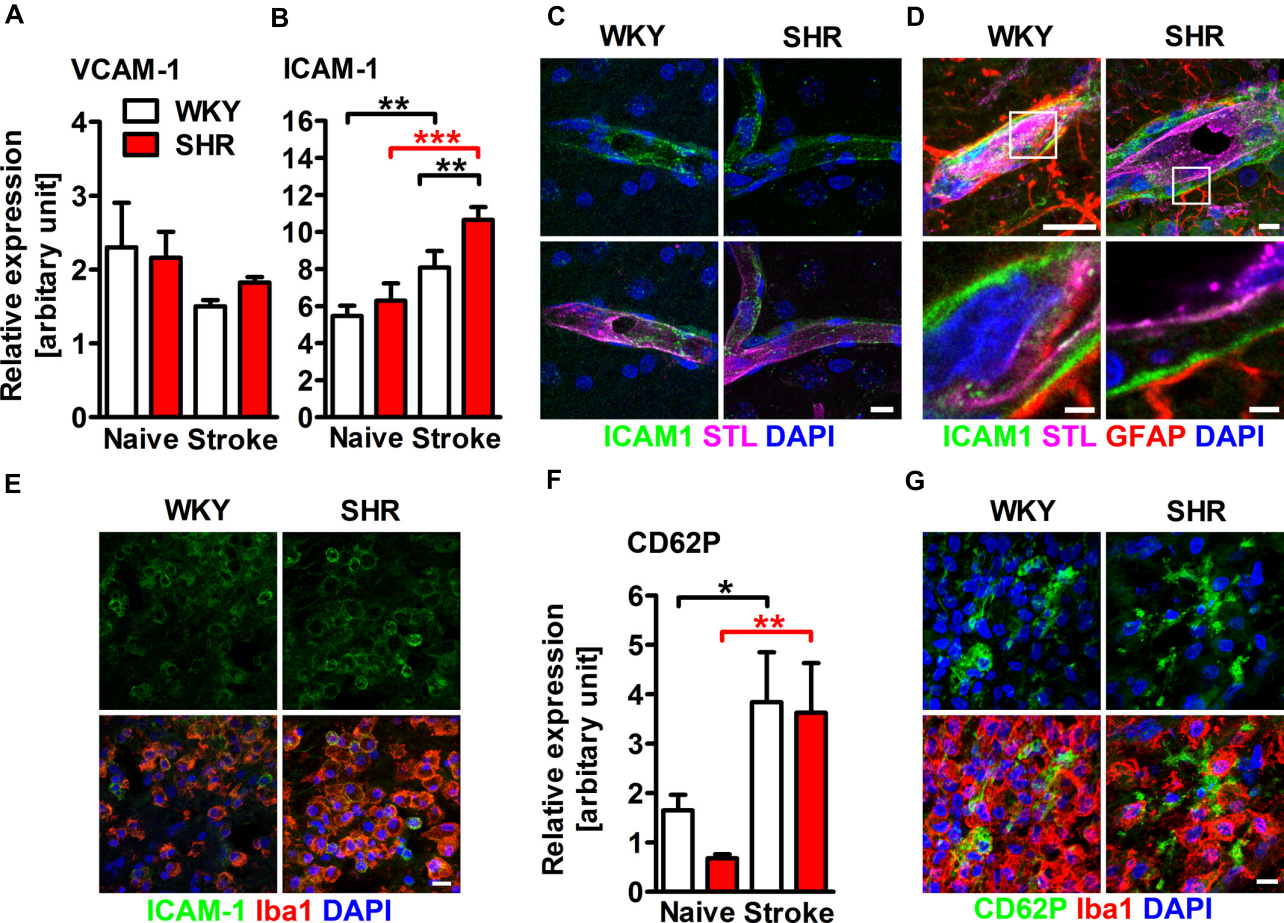
**Adhesion molecule expression within the ischemic brain of SHRs and WKYs assessed by quantitative real time PCR and immunofluorescence**. mRNA expression of **(A)** vascular cell adhesion molecule 1 (VCAM-1) and **(B)** intercellular adhesion molecule 1 (ICAM-1) mRNA expression. **(C–E)** Co-staining of ICAM-1 with *Solanum tuberosum* lectin (STL), glial fibrillary acidic protein (GFAP) and ionized calcium-binding adapter molecule 1 (Iba1). ICAM-1 is co-expressed by STL+ endothelial cells within the ipsi- and contralateral hemisphere **(C)**, within the parenchymal basement membrane of post-capillary venules adjacent to the ischemic lesion **(D)** and by Iba1+ myeloid leukocytes within the ischemic lesion **(E)**. mRNA expression of **(F)** P-selectin (CD62P) and-staining of CD62P and Iba1 **(G)**. Nuclei were counterstained with DAPI; *n* = 3/3/3/3, data are mean values ± standard deviation. ^∗^*p* < 0.05, ^∗∗^*p* < 0.01, ^∗∗∗^*p* < 0.001. Scale bars: **(C)** 10 μm; **(D)** 10 and 1.5 μm; **(E)** 10 μm; **(G)** 10 μm.

Adhesion molecules are crucial for the recruitment of leukocytes into the ischemic lesion. We therefore aimed to investigate whether hypertension and stroke influences the expression of AMs in the brain. qRT-PCR of whole hemispheric lysates revealed comparable mRNA expression of VCAM-1 in naive and stroked SHR and WKY (**Figure 2A**). The expression of ICAM-1 mRNA was also comparable in naive SHR and WKY. PT caused a significantly increased ICAM-1 expression in both rat strains, but this effect was significantly higher in SHR (**Figure 2B**). Immunofluorescence staining showed that ICAM-1 was expressed in small capillaries throughout the ipsi- and contralateral hemisphere (**Figure 2C**) and within the parenchymal basement membrane of post-capillary venules adjacent to the ischemic lesion (**Figure 2D**). Interestingly, we also found ICAM-1 expression on round, Iba1+ leukocytes within the ischemic lesion (**Figure 2E**) and rarely within the lesion border (not shown). Together, these observations indicate that the increased expression of ICAM-1 mRNA in the ischemic hemisphere primarily derives from the vasculature and from infiltrating leukocytes. PT caused a distinct increase of P-selectin (CD62P) expression in both SHR and WKY, a finding that was not further influenced by hypertension (**Figure 2F**). Immunofluorescence staining revealed strong CD62P expression on vessels within the ischemic lesion (not shown). We furthermore observed spotty CD62P expression without associated nuclei surrounding Iba1+ leukocytes within the ischemic lesion (**Figure 2G**), possibly corresponding to CD62P expressing platelets.

**FIGURE 3:**
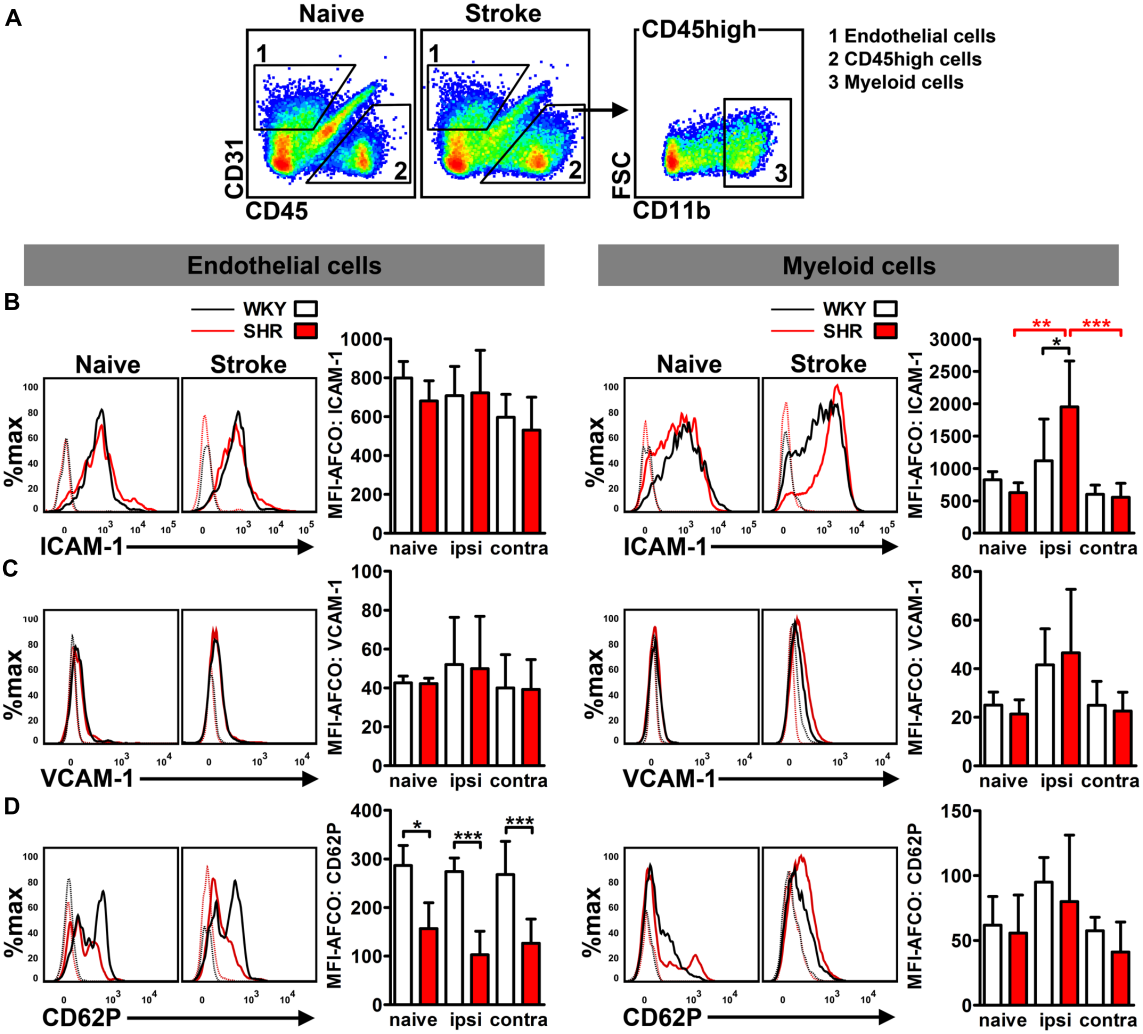
**Adhesion molecule expression on brain endothelial cells and infiltrated CD45 high leukocytes was determined by flow cytometry. (A)** Gating strategy to differentiate CD31+ endothelial cells (1) from CD11b+ myeloid leukocytes (3) in whole hemispheric lysates. Measurement of median fluorescence intensities (MFIs) of **(B)** intercellular adhesion molecule 1 (ICAM-1), **(C)** vascular cell adhesion molecule 1 (VCAM-1) and **(D)** P-selectin (CD62P) on surfaces of endothelial cells and myeloid leukocytes in brains of SHRs and WKYs; *n* = 3/3/4/6, data are mean values ± standard deviation. ^∗^*p* < 0.05, ^∗∗^*p* < 0.01, ^∗∗∗^*p* < 0.001. AFCO, autofluorescence control; ipsi, ipsilateral; contra, contralateral.

To differentiate AM expression on cerebral endothelial cells from infiltrated CD45 highly positive (CD45++) leukocytes, we performed flow cytometry analysis of CD31, CD45, and CD11b expression in whole hemispheric lysates (**Figure 3A**). Analysis of MFIs of ICAM-1, VCAM-1, and CD62P allowed us to quantify the average expression of these AMs on brain endothelial cells (CD31+/CD45-) and myeloid leukocytes (CD31-/CD45++/CD11b+). Neither hypertension nor stroke had an impact on the endothelial expression of ICAM-1 and VCAM-1 at day 4 after PT (**Figures 3B,C**). By contrast, we found a significantly decreased expression of CD62P on brain endothelial cells of naive SHR, a finding that was not further influenced by stroke (**Figure 3D**). Leukocyte AM expression was not influenced by hypertension in naive rats, but by stroke: SHR exhibited a significantly increased MFI of ICAM-1 on myeloid leukocytes (**Figure 3B**), whereas VCAM-1 and CD62P remained unchanged (**Figures 3C,D**). Taken together, these data suggests that the increased ICAM-1 expression on innate immune cells in SHR may facilitate leukocyte transmigration by intravascular leukocyte–leukocyte interactions. By contrast, the brain endothelium of SHR is not increasingly permissive for transmigrating leukocytes.

### Post-stroke Chemokine Expression in the Brain is Altered by Hypertension

**FIGURE 4:**
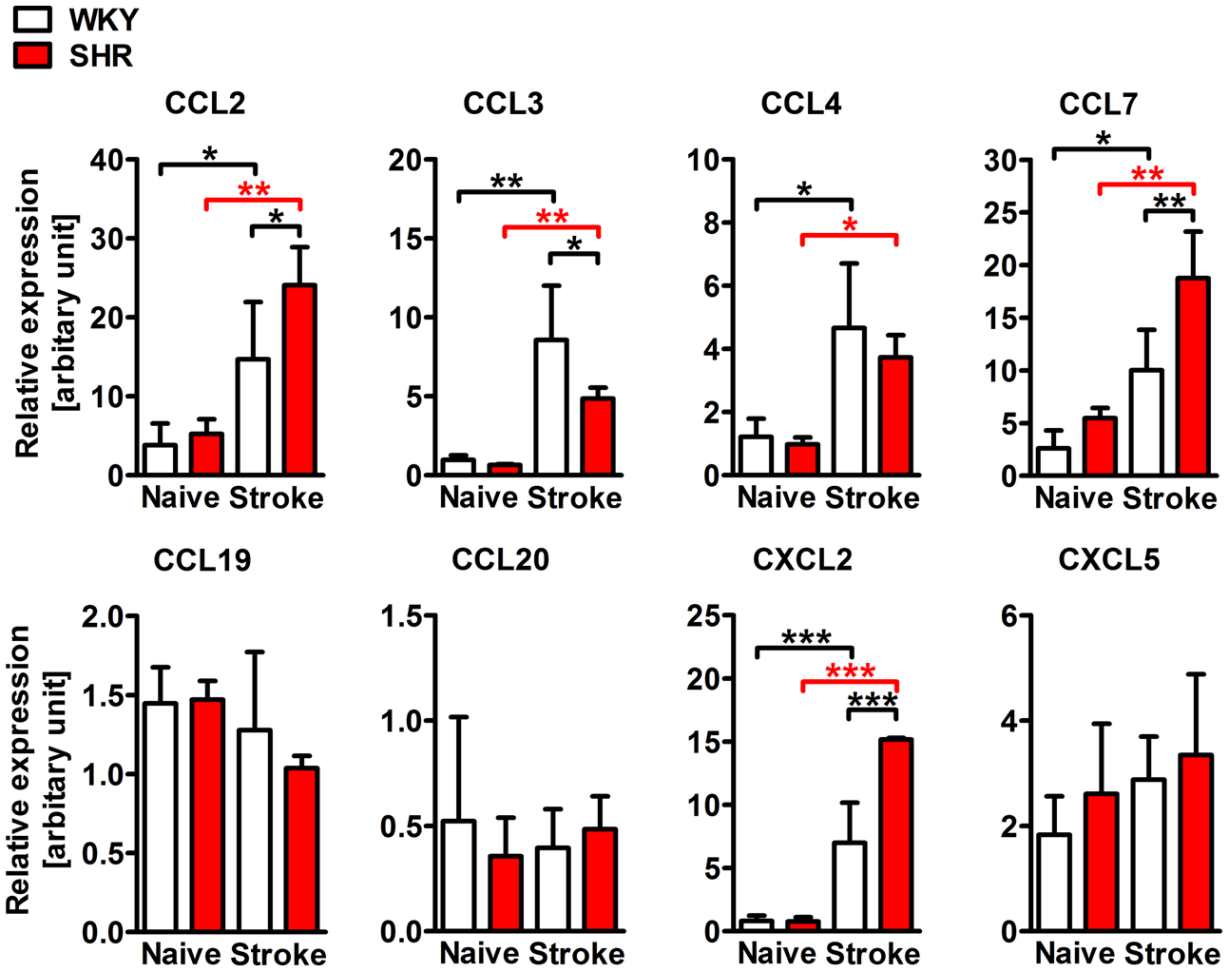
**mRNA expression of the CC chemokine ligands 2 (CCL2), 3 (CCL3), 4 (CCL4), 7 (CCL7) and of the CXC chemokine ligands 2 (CXCL2) and 5 (CXCL5) in whole hemispheric lysates of SHRs and WKYs**. *n* = 3/3/3/3, data are mean values ± standard deviation. ^∗^*p* < 0.05, ^∗∗^*p* < 0.01, ^∗∗∗^*p* < 0.001.

We next asked whether the stroke-induced expression of chemokines is also altered by pre-existing hypertension. To answer this question, we examined mRNA expression of selected CC and CXC chemokine ligands in whole hemispheric lysates. Naive SHR and WKY showed comparable levels of CC and CXC chemokines. However, the expression of CCL2, CCL3, CCL4, CCL7, and CXCL2 was strongly increased after stroke, whereas CCL19, CCL20, and CXCL5 expression levels were not changed (**Figure 4**). When comparing SHR and WKY subjected to stroke, we observed a significantly higher expression of CCL2, CCL7, and CXCL2 in SHR. By contrast, CCL3 was significantly decreased in SHR (**Figure 4**).

### Post-stroke Inflammation is Increased in SHR

**FIGURE 5:**
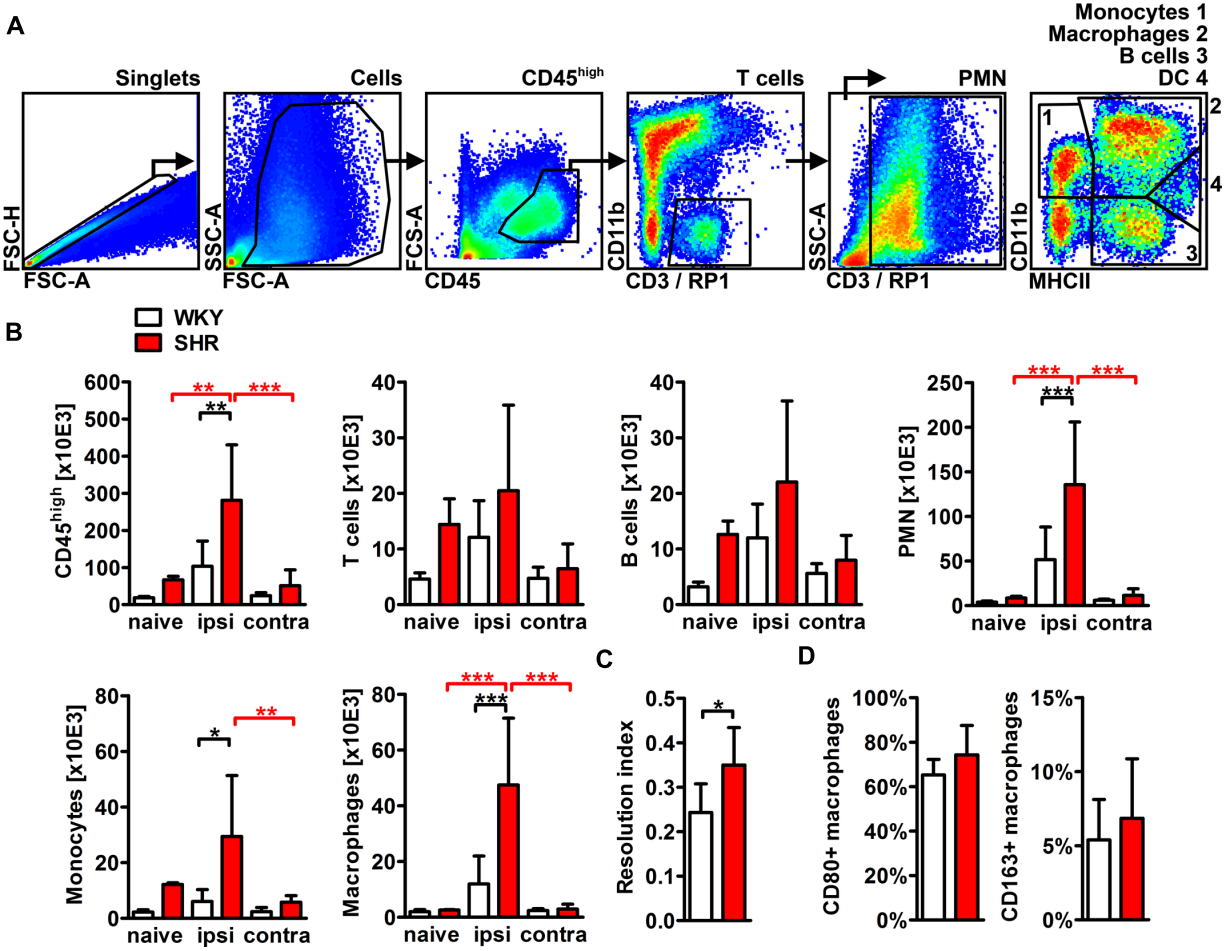
**Differentiation and quantification of leukocyte subsets in the brain of SHRs and WKYs subjected to photothrombotic stroke. (A)** Gating strategy to assess CD45 high positive leukocytes, T cells, B cells, polymorphonuclear granulocytes (PMN), monocytes, macrophages and CD11b+ dendritic cells in whole hemispheric lysates. **(B)** Absolute cell counts of the aforementioned leukocytes subsets were determined by Trucount measurements. **(C)** The resolution index was calculated by dividing the PMN count through the macrophage count. **(D)** M1 or M2 macrophage polarization was determined by macrophage expression of CD80 or CD163, respectively; *n* = 3/3/5/8, data are mean values ± standard deviation. ^∗^*p* < 0.05, ^∗∗^*p* < 0.01, ^∗∗∗^*p* < 0.001. DC, dendritic cells; ipsi, ipsilateral; contra, contralateral.

**FIGURE 6:**
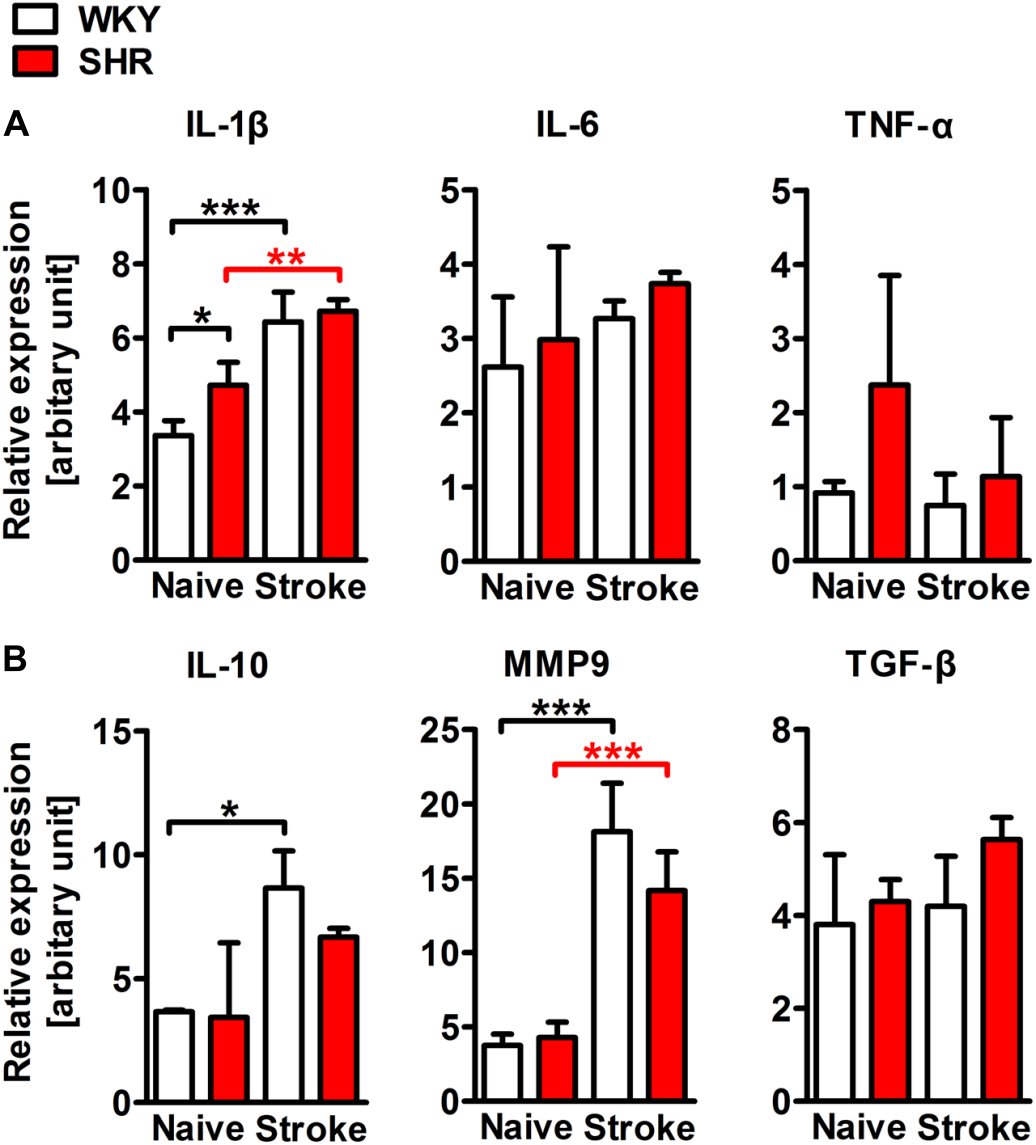
**mRNA expression of representative M1 (IL-1β, IL-6, and TNF-α) and M2 (IL-10, MMP9, and TGF-β) macrophage markers in whole hemispheric lysates of SHRs and WKYs; *n* = 3/3/3/3, data are mean values ± standard deviation**. ^∗^*p* < 0.05, ^∗∗^*p* < 0.01, ^∗∗∗^*p* < 0.001. Scale bars: **(A)** 200 μm; **(B)** 20 μm.

To characterize and quantify the inflammatory infiltrate within the ischemic brain, we performed flow cytometric analyses of whole hemispheric lysates. The antibody panel used in this study allowed us to differentiate T cells, B cells, PMN, monocytes, macrophages, and CD11b+ dendritic cells (**Figure 5A**). None of the investigated leukocyte subsets differed between naive SHR and WKY. However, stroke caused a fourfold increase of CD45 highly positive cells that infiltrated into the ischemic lesion in SHR, while we found only a minor, statistically not significant increase in WKY (**Figure 5B**). T cells, B cells (**Figure 5B**) and CD11b+ dendritic cells (not shown) were not altered by stroke. In contrast, we found that primarily PMN, monocytes, and macrophages gave rise for the significant increase of CD45 high leukocytes in SHR. Each of the three cell populations was significantly increased after stroke in SHR when compared to naive SHR or WKY with stroke (**Figure 5B**). The calculation of the resolution index, the ratio between macrophages and PMN, allows an estimation of the progress of the clearance of inflammation (Bratton and Henson, 2011). Interestingly, this index was significantly increased in SHR indicating that the inflammation started earlier or was cleared more rapidly in SHR. Macrophages participating in inflammatory processes can be differentiated by distinct functional properties with the M1 and M2 phenotypes being the extremes. Flow cytometric analysis revealed similar proportions of M1 (CD80+) and M2 (CD163+) macrophages in ischemic hemispheres of SHR and WKY (**Figure 5D**). This finding was further corroborated by the qRT-PCR analysis of the typical M1 markers IL-1β, IL-6, and TNF-α as well as M2 markers IL-10, MMP9, and TGF-β (Courties et al., 2014), which were comparable between SHR and WKY after stroke (**Figure 6**). Together, our findings provide evidence that arterial hypertension strongly augments the innate immune response to stroke, but has no impact on macrophage polarization within the ischemic lesion.

## Discussion

For decades, stroke has been considered a strictly localized neurological disease with a loss of neural tissue entailing classical stroke-related symptoms. However, ischemic tissue damage always causes a sterile inflammatory response that is indispensable for controlling brain repair and regeneration, but also strongly contributes to secondary damage and functional deterioration (Iadecola and Anrather, 2011; Gelderblom et al., 2012; Shichita et al., 2012a; Neumann et al., 2015).

In the present study, the infarct volume was comparable between WKY and SHR at day 1, but significantly higher in SHR 3 days after PT. The final lesion size in this stroke model is reached 4 h after ischemia onset (Grome et al., 1988), thus later changes may be attributed to the brain water content, delayed cell death or secondary inflammatory mechanisms (Witte and Stoll, 1997; Carmichael, 2005; Shichita et al., 2012b). Brain edema developed comparably in WKY and SHR, but the infarct volume strongly correlated with the amount of CD45 highly positive leukocytes in the ischemic hemisphere.

As described previously (Gronberg et al., 2013; Lehmann et al., 2014; Moller et al., 2014) and in accordance with prototypic models of sterile inflammation (Bratton and Henson, 2011), the early inflammatory infiltrate primarily consisted of PMNs, monocytes, and macrophages whereas T- and B cell counts were not changed 4 days after stroke. Short-lived PMNs constitute the first wave of immune cells that invade into the ischemic brain, where they strongly contribute to brain damage (Akopov et al., 1996; Price et al., 2004; Gelderblom et al., 2012; Jickling et al., 2015; Neumann et al., 2015). In fact, increased PMN infiltration could be one conclusive explanation for the larger infarct sizes in SHR. Apoptotic neutrophils are phagocytosed by monocytes and macrophages which is, in turn, an important impulse for the resolution of necrotic tissue and the initiation of macrophage repair and regeneration programs (Chu et al., 2015; Headland and Norling, 2015; Ritzel et al., 2015). SHR exhibited significantly higher monocyte and macrophage counts in the ischemic hemisphere, which might be a consequence of the preceding increase of neutrophil infiltration. The relative shift toward macrophages (Resolution Index, **Figure 5C**) in SHR might argue for an accelerated resolution of inflammation in hypertensives, but this finding was not supported by the investigation of M1/M2 macrophage polarization markers which were comparable between WKY and SHR. Future studies focusing on efferocytosis as the key event of inflammation resolution in SHR and normotensive controls are needed to clarify this issue.

The correct timing and balance between protective and detrimental inflammation is regulated by four major categories of mechanisms: (1) the amount and activation state of circulating immune cells and their successive replenishment from bone marrow and spleen (Leuschner et al., 2012; Courties et al., 2015); (2) Chemoattractants secreted by parenchymal and endothelial cells of the ischemic brain (Mirabelli-Badenier et al., 2011); (3) AM expression on cerebral capillaries and post-capillary venules (Yilmaz and Granger, 2008); (4) The environmental cues within the ischemic brain tissue that adjust and confine successive immune responses (Iadecola and Anrather, 2011; Hu et al., 2012). Cumulating evidence suggests that hypertension affects at least some of the aforementioned mechanisms and may thus increase detrimental neuroinflammation and aggravate stroke severity.

An impaired autonomic response is one unifying symptom of neurogenic hypertension in SHR and patients (Mancia and Grassi, 2014; Zubcevic et al., 2014). Increased adrenergic output, e.g., by arterial hypertension has been shown to have a direct effect on the bone marrow hematopoietic niche resulting in increased levels of monocytes and neutrophils in the circulation (Heidt et al., 2014; Zubcevic et al., 2014). In fact, this and previous studies (Schmid-Schonbein et al., 1991) showed increased monocyte and neutrophil counts in the blood of naive SHR, which may eventually contribute to increased immune cell infiltration and infarct expansion in the ischemic brain. Compatible with this, a strong positive correlation between circulating neutrophils and infarct volume has been described in stroke patients (Buck et al., 2008).

In hypertensives, the brain microvasculature is subjected to a continuous pulsatile barotrauma which causes the remodeling of the vessel walls and endothelial dysfunction (Wang et al., 2004; Scuteri et al., 2011; Yannoutsos et al., 2014). Increased expression of AMs and chemokines are hallmarks of endothelial activation (Tummala et al., 1999; Schober and Zernecke, 2007; Liao, 2013) promoting recruitment of circulating immune cells into the brain (Ley et al., 2007). Patients with arterial hypertension and cerebral small vessel disease showed increased levels of both soluble selectins (CD62P and CD62E) and integrins (ICAM-1 and VCAM-1; Sanada et al., 2005; Shalia et al., 2009; Rouhl et al., 2012) which can be considered as surrogates for their endothelial expression (Zonneveld et al., 2014). We recently found that VCAM-1 expression was significantly increased in cerebral endothelial cells of middle-aged SHR (Kaiser et al., 2014). We therefore hypothesized that increased endothelial AM expression due to hypertension may augment leukocyte adhesion and transmigration after stroke. However, in this study, we did not observe increased AM expression in endothelial cells of naive SHR possibly due to their relatively young age (12–14 weeks). By contrast, we found significantly lower endothelial CD62P expression in SHR which may be a consequence of enhanced proteolytic cleavage by circulating metalloproteinases (Chen et al., 2012). PT induced a strong upregulation of CD62P expression in both WKY and SHR, but this was neither attributed to endothelial cells nor infiltrating immune cells. Alternatively, immunohistochemistry revealed diffuse extracellular CD62P expression within the infarction which may represent platelets (Yang et al., 2009) crosslinking infiltrating leukocytes (Franks et al., 2010; Mezger et al., 2015).

Similar to CD62P, ICAM-1 expression was significantly increased in ischemic hemispheres of WKY and, even more, SHR. Flow cytometry further specified an upregulation of ICAM-1 on infiltrating myeloid cells of SHR. In contrast, ICAM-1 expression on endothelial cells was not changed 4 days after stroke, possibly because responsive endothelial AM expression peaks earlier (Jander et al., 1995; Yilmaz and Granger, 2008). Leukocytic ICAM-1 expression occurs primarily on monocytes/macrophages and enhances endothelial transmigration by homotypic leukocyte–leukocyte interactions (Goebeler et al., 1993; Meisel et al., 1998; Ruetten et al., 1999; Steidl et al., 2000; Novosad et al., 2011). ICAM-1 expression on antigen presenting cells may further act as an important costimulatory signal for T cell activation at the site of inflammation (Goebeler et al., 1993; Real et al., 2004). The fact that leukocytes also express functionally relevant ICAM-1 provides novel approaches to interpret findings from stroke studies using ICAM-1 antibodies or ICAM-1 knock-out animals (Connolly et al., 1996; Enlimomab Acute Stroke Trial Investigators, 2001). At the moment, we do not have a conclusive explanation for the increased levels of ICAM-1 on myeloid immune cells of SHR. One could speculate that the known pro-inflammatory state in hypertensives increases leukocyte ICAM-1 expression and thus contributes to increased post-stroke inflammation, but this assumption warrants further investigations.

Leukocyte trafficking into the ischemic brain is regulated by various chemokines that attract leukocytes toward the affected vasculature and support AM-regulated leukocyte transmigration (Ley et al., 2007). Most of the inflammatory chemokines were found to be upregulated after stroke and are secreted either by brain resident astrocytes, neurons, microglia, endothelial cells, or by infiltrating leukocytes (Mirabelli-Badenier et al., 2011). The relevance of post-stroke chemokine secretion has been recently underpinned by a study showing that the neutralization of circulating chemokines using a broad spectrum chemokine binding protein caused a significant reduction of leukocytes infiltrating the ischemic brain (Lee et al., 2015). In this study, PT induced a strong increase of chemokines that attract monocytes (CCL2, CCL3, CCL4, and CCL7) and neutrophils (CXCL2), whereas T cell-attracting chemokines (CCL19, CCL20) were not altered. This pattern adequately reflects the composition of the ischemic brain infiltrate at this time point, as discussed above. While the expression levels in naive animals did not differ, CCL2, CCL7, and CXCL2 expression was significantly amplified in SHR after stroke. This could be interpreted as both, a cause or consequence of increased leukocyte infiltration in SHR. Interestingly, we observed a contrary finding for CCL3 that was expressed at significantly higher levels in ischemic hemispheres of WKY. It has been reported in experimental autoimmune encephalomyelitis that mononuclear cell infiltration in the acute disease and the relapse phase is controlled by CCL3 or CCL2, respectively (Kennedy et al., 1998), suggesting a distinct functional differentiation of both chemokines. This idea is further corroborated by the recent finding that CCL3 deficiency strongly increases inflammatory cell infiltration after experimental traumatic brain injury (Israelsson et al., 2014). We therefore hypothesized that the unexpected decrease of CCL3 expression in SHR could be attributed to the accelerated resolution of inflammation as corroborated by an increased resolution index (**Figure 5C**).

Our study has limitations. The use of SHR and WKY rat strains may be confounded by genetic differences apart from or primary to hypertension (Zhang-James et al., 2013). Thus, it is difficult to clearly differentiate between pathophysiological changes due to the hypertensive state or other unknown phenotypes. One example is the tight junction and adhesion molecule (JAM-1) which is already increased in brains of pre-hypertensive SHR and contributes to the development of hypertension. On the other hand, it also mediates leukocyte adhesion and platelet aggregation and may thus contribute to the course of post-stroke inflammation (Waki et al., 2007). Moreover, we focused our analyses on peripheral CD45 highly positive leukocytes even though microglia and astrocytes also significantly contribute to stroke pathophysiology and may be functionally altered by arterial hypertension (Marks et al., 2001; Yamagata, 2012). Immune cell depletion studies would be necessary to separate the pathophysiological relevance of certain immune cell subsets from brain-resident cells under hypertensive conditions. The study is finally limited by a relatively small sample size that may have caused Type II errors especially in the behavioral tests that empirically underlie increased variability.

In summary, we observed that pre-existing hypertension caused larger stroke sizes possibly as consequence of a profound increase of post-stroke inflammation. This finding could be explained by increased numbers and activation status of circulating myeloid leukocytes and increased levels of leukocyte-attracting chemokines in hypertensives. Future translational stroke research should consider the immunological effects of the frequent co-morbidity hypertension, especially when targeting the immune response to stroke.

## Author Contributions

KM, CP, JB, GW, and D-CW designed and supervised the project. KM, CP, AK, IS, JS, and ND performed the experiments and analyzed the data. KM, GW, and D-CW wrote the manuscript.

## Conflict of Interest Statement

The authors declare that the research was conducted in the absence of any commercial or financial relationships that could be construed as a potential conflict of interest.
